# Potential Novel Food-Related and Biomedical Applications of Nanomaterials Combined with Bacteriocins

**DOI:** 10.3390/pharmaceutics13010086

**Published:** 2021-01-11

**Authors:** Atanu Naskar, Kwang-sun Kim

**Affiliations:** Department of Chemistry and Chemistry Institute for Functional Materials, Pusan National University, Busan 46241, Korea; atanunaskar@pusan.ac.kr

**Keywords:** bacteriocin, nanomaterial, biomedical applications, nanomedicine, bacteriocin nanoconjugate

## Abstract

Bacteriocins are antimicrobial peptides or proteinaceous materials produced by bacteria against pathogens. These molecules have high efficiency and specificity and are equipped with many properties useful in food-related applications, such as food preservatives and additives, as well as biomedical applications, such as serving as alternatives to current antibacterial, antiviral, anticancer, and antibiofilm agents. Despite their advantages as alternative therapeutics over existing strategies, several limitations of bacteriocins, such as the high cost of isolation and purification, narrow spectrum of activity, low stability and solubility, and easy enzymatic degradation, need to be improved. Nanomaterials are promising agents in many biological applications. They are widely used in the conjugation or decoration of bacteriocins to augment the activity of bacteriocins or reduce problems related to their use in biomedical applications. Therefore, bacteriocins combined with nanomaterials have emerged as promising molecules that can be used in various biomedical applications. This review highlights the features of bacteriocins and their limitations in biomedical applications and provides a detailed overview of the uses of different nanomaterials in improving the limitations. Our review focuses on the potential applications of nanomaterials combined with bacteriocins as new designer molecules for use in future therapeutic strategies.

## 1. Introduction

Bacteriocins are a group of ribosomally synthesized peptides that are secreted extracellularly by various gram-positive and gram-negative bacteria [1], although a majority of bacteriocins reported are produced by the former, especially lactic acid bacteria (LAB) [2,3]. Extensive studies have been carried out on bacteriocins owing to their excellent antibacterial activity, which is closely associated with strain producing species. In addition, bacteriocins have garnered considerable research attention in the field of biomedicine owing to their generally recognized as safe (GRAS) status, and because they are safe for human consumption due to their degradation by gastrointestinal proteases [4]. They are also being modified to improve the antibacterial spectrum. Most of the well-known bacteriocins are produced by gram-positive bacteria, whereas only a few from gram-negative bacteria have been characterized [5]. The activity of these small bacteriocins consisting of cationic molecules (30–60 amino acids) vary throughout the antibacterial spectrum, mainly due to their amphiphilic helices.

Bacteriocins are widely used as natural food preservatives—substances that delay the growth of microorganisms—in the food industry, because they are easily degraded by enzymes produced in the human gastrointestinal tract [4]. The high quality and safety profile of bacteriocins as natural food preservatives is possible without the use of chemical preservatives, which is strictly regulated by governmental agencies, such as the Food and Drug Administration (FDA) in the United States, owing to their safety issues. Generally, bacteriocins can be directly added to food or incorporated into food during cultivation with the help of bacteriocin-producing bacterial strains. More recently, bacteriocins have gained considerable attention in the healthcare industry as antibacterial and anticancer agents [6]. Some bacteriocins, such as nisin, have shown excellent and specific antibacterial activity against multi-drug resistant (MDR) strains of gram-positive bacteria, such as methicillin-resistant *Staphylococcus aureus* (MRSA) [7]. Moreover, gram-negative bacteria are naturally resistant to the action of bacteriocins produced by gram-positive bacteria, which are widely explored in foods. It is worthy to note that the bacteriocins produced by gram-positive bacteria can be combined with antibacterial nanoparticles to inhibit the growth of gram-negative bacteria such as *P. aeruginosa*. In this respect, Au-nisin successfully experimented for the bacterial growth inhibition of *E. coli* and *P. aeruginosa* [8]. Currently, researchers are investigating the biomedical properties of bacteriocins in several anticancer studies and have reported promising results [9]. Therefore, researchers are highly interested to use bacteriocins as safe materials in biomedical applications.

Despite their promising advantages, bacteriocins have certain limitations that hinder their applications in the field of biomedicine [10]. These limitations include the following: (i) easy degradation by proteolytic enzymes, (ii) constricted antibacterial spectrum, (iii) requirement of high dosage to kill MDR bacteria, (iv) high production cost, and (v) low natural production yield. Based on these limitations, only three FDA-approved bacteriocins (nisin, pediocin, and Micocin^®^) are available for food preservation and anti-spoilage processes [11]. The optimization of different production conditions, purification methods, and combinations with other antimicrobial agents or antibiotics has been assessed by various studies to overcome the aforementioned shortcomings in ensuring the broad application of bacteriocins in biomedicine. As adjuvant bioactive materials for bacteriocins, nanomaterials seem highly promising to overcome these challenges [12]. The major reasons for the use of nanomaterials can be attributed to their following characteristics: (i) broad antibacterial spectrum, (ii) stability in physiological solution, (iii) high surface area-to-volume ratio, (iv) easy to synthesize with low production cost, and (v) non-toxic nature at low concentrations. Bacteria are unable to generate rapid resistance against nanomaterials owing to their multi-dimensional approach compared to antibiotics, which targets a single cellular component [13]. Therefore, the combination of bacteriocins and nanomaterials offers new tactics to overcome the limitations posed by the sole use of bacteriocins in biomedical applications. In this review, we will focus on the advantages of bacteriocin–nanomaterial combinations in not only food-related applications, but also antibacterial and other biomedical applications.

## 2. Different Bacteriocins and Their Spectrum

It is necessary to determine the different types of bacteriocins based on their activity against closely related species. Generally, limited studies on bacteriocins from gram-negative bacteria have been conducted owing to their narrow spectrum of activity against closely related species [5]. Comparatively, the effect of gram-positive bacteriocins is much broader, and therefore, it has been studied extensively. 

Gram-negative bacteriocins: One of the representative bacteriocins from gram-negative bacteria is colicin from *Escherichia coli.* It is a high molecular-weight protein that is widely used to control various gram-negative bacteria [14]. Another bacteriocin produced by *E. coli* is microcin, which is similar to gram-positive bacteriocins in terms of its pH and thermal stability along with protease resistance [15]. In addition, other gram-negative bacteria produce different bacteriocins (e.g., pyocins or aeruginocins from *P. aeruginosa* and klebicin from *Klebsiella pneumonia*) [5]. The limited spectrum of these gram-negative bacteriocins at times can be advantageous, since these bacteriocins can be used depending on a particular situation or for a specific purpose. However, this narrow antibacterial spectrum can be resolved with the use of nanomaterials, which will be discussed in later sections.

**Figure 1:**
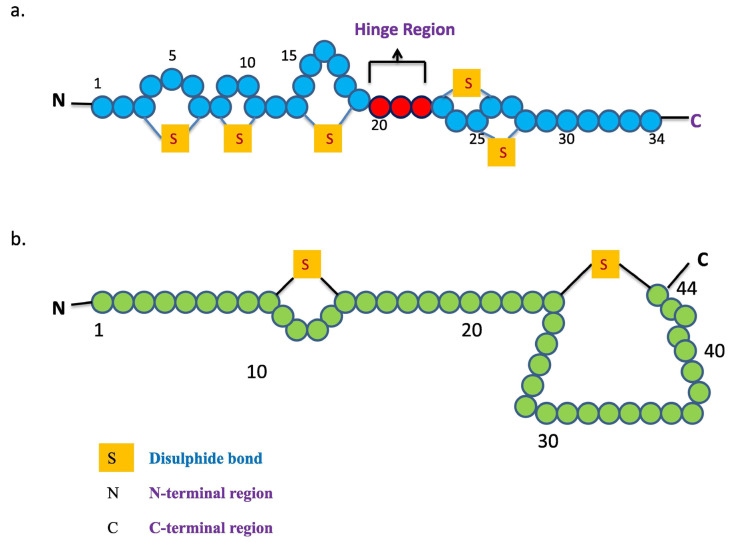
Primary structure of (**a**) nisin and (**b**) pediocin divided into N-terminal and C-terminal. Reproduced with. permission from Ref. [16], copyright 2019, Elsevier.

Gram-positive bacteriocins: Gram-positive bacteriocins possess broad antibacterial spectrum compared to gram-negative bacteriocins. The large spectrum is mainly attributed to the presence of a thick multilayered peptidoglycan wall instead of an outer membrane. This outer organization enables the penetration of small peptides without any receptor binding [5]. Gram-positive bacteriocins are largely produced by LAB. LAB bacteriocins are extensively used, because they have been accorded GRAS status by the U.S. FDA. Nisin and pediocin are well-known bacteriocins produced by gram-positive bacteria [16] and are already approved for commercial use by the FDA. Nisin, which is synthesized as a precursor peptide produced by *Lactococcus lactis*, contains 57 amino acids and has a molecular weight (MW) of 3354 daltons. Its mature form of 34 amino acids is further generated by post-translation modifications (Figure 1a). However, unlike nisin, pediocin PA-1 is produced as a mature form of 44 amino acids with MW of 4646.95 daltons and is not further processed by post-translation modification (Figure 1b).

## 3. Stability of Bacteriocins

Bacteriocins have potential biomedical applications considering their antibacterial activity *in vitro* [7,16,17,18,19]. They have a low *in vivo* stability and are vulnerable to proteolytic enzyme degradation, which hinders their clinical applicability [10]. Furthermore, bacteriocins are labile in tissues, serum, and organs (such as the liver and kidneys). They thus need to be modified or functionalized to ensure stability in physiological solutions. The stability of nisin, a frequently used variant for biomedical applications as an antibacterial agent or food preservative, is dependent on the environmental pH [20]. For instance, solubilized nisin is active and stable in acidic pH, whereas its solubility decreases in alkaline pH, which hinders its biological activity. Different functionalization methods have been reported to address stability for bacteriocins; cyclization by the incorporation of d-amino acids is one such process [21]. The stability of nisin (i.e., NisB and NisC) can also be increased by modifying unrelated peptides via the modification machinery used for the incorporation of lanthionine ring structures in nisin [22]. Peng et al. [23] enhanced the stability and bactericidal activity of nisin by conjugation with gellan gum. The resulting gellan–nisin conjugate exhibited excellent pH, heat, and chymotrypsin resistance compared with those of unmodified nisin. Similarly, Bagde et al. [24] reported enhanced bacteriocin stability via immobilization into cellulose nanocrystals. The bacteriocins extracted from *Enterococcus faecium* and immobilized on cellulose nanocrystals exhibited a 50% increase in stability. In another study, layered double hydroxide nanoparticles were used to improve the stability of the bacteriocin avicin A, which also enhanced its antibacterial activity [25]. Liposomes have also been used to encapsulate bacteriocins to make them more stable [26]. Malheiros et al. [27] reported the use of nanovesicles to encapsulate bacteriocin-like substances for enhanced stability and antilisterial activity. Chitosan nanoparticles are used to a considerable extent by many researchers to stabilize and enhance the biological activity of bacteriocins [28]. Nisin has also been encapsulated with poly-l-lactide (PLA) nanoparticles for potent and stable release of bacteriocins in food preservation [29]. Therefore, it is clear that future *in vivo* applications of bacteriocins are possible upon improvement in their stability via the use of nanomaterials.

## 4. Advantages and Limitations of Bacteriocins

Bacteriocins have several properties that render them advantageous for various biomedical applications; these include as antibacterial agents, drug delivery agents, and functional foods [6,21]. In terms of antibacterial activity, they present low toxicity to eukaryotic cells, stability in physiological solutions after functionalization, stability at high temperatures, and low minimum inhibitory concentrations against numerous bacterial strains. Bacteriocins also possess selective antimicrobial activity [30]. Thus, they are regarded as promising alternatives to [21] or synergistic materials for [31] currently used antibiotics. The synergistic property of bacteriocins allows lower usage of the antibiotic agent, thereby reducing in the risk of cytotoxic effects arising from its use. Furthermore, bacteriocins are generally protected from the acquisition of bacterial resistance. Therefore, it has the potential to reduce the development of MDR strains, as bacteriocins can be used as antibacterial agents either alone or together with nanomaterials instead of antibiotics. Bacteriocins capped with silver nanoparticles have reduced cytotoxicity [32]. Bacteriocins are also used for food preservation owing to their impressive *in vitro* and in situ effectiveness against various food-borne and human pathogens [4]. Three bacteriocins, nisin, pediocin, and Micocin^®^, have been approved by the FDA for use as food preservatives and antispoilage agents [11]. In terms of other biomedical applications, bacteriocins were effectively used for therapeutic drug delivery in anticancer treatment [6]. In this regard, Joo et al. [33] reported that nisin at low concentration (2.5%) effectively induces preferential apoptosis combined with cell-cycle arrest. It also reduces cell proliferation in head and neck squamous-cell carcinoma. Owing to these advantageous properties, bacteriocins have various biomedical applications and are used in catheter coatings [34], oral tablets [35], chewing gum [36], aquaculture dry sprays [37], hydrogels [38], scaffolds [39], and food packaging [40].

Despite the various potential biomedical applications of bacteriocins, their actual clinical use is limited. The primary issue is similar to that for antibiotics—bacteria can develop resistance against bacteriocins [41]. This needs to be resolved immediately and completely for its *in vivo* antibacterial applications to be feasible. The combination of nanoparticles with bacteriocins has the potential to resolve this issue, because bacteria do not develop rapid resistance against nanomaterials [13]. Another limitation of bacteriocins is their degradation by proteases [10]. Most reported bacteriocins are sensitive to proteases; they are rapidly degraded by proteases such as proteinase K and pepsin. This should be resolved for effective targeted drug delivery or use as antibacterial agents. The encapsulation of bacteriocins with nanomaterials is an effective way to resolve this issue [10,16]. Additionally, the toxicity of bacteriocins, specially nisin and pediocin, is debatable [42]. However, they are harmless for humans and animals when used as food preservatives. Overall, the limitations of bacteriocins need to be resolved to realize their immense potential for biomedical applications.

## 5. Bacteriocin–Nanomaterial Combination

As discussed in previous sections, certain limitations need to be resolved before bacteriocins can be used for various *in vivo* biomedical applications. These limitations include the following: (i) tendency towards degradation by proteolytic enzymes, (ii) limited antibacterial spectrum, (iii) failure to prevent the development of bacterial resistance, as with currently used antibiotics, and (iv) high production cost with low yield [10].

Nanomaterials have been recently used to potentially overcome such limitations [10,16]. For instance, researchers have already developed bacteriocin–nanomaterial complexes or bacteriocin–nanoconjugates for various biomedical applications of bacteriocins. Multiple advantages for the use of bacteriocin–nanoconjugates have been reported: (i) increased stability for long periods of use, (ii) protection from proteolytic enzyme degradation, and (iii) synergistic activity. The potential applicability of bacteriocin–nanoconjugates with different nanomaterials has been described in following sections.

### 5.1. Liposomes

**Figure 2:**
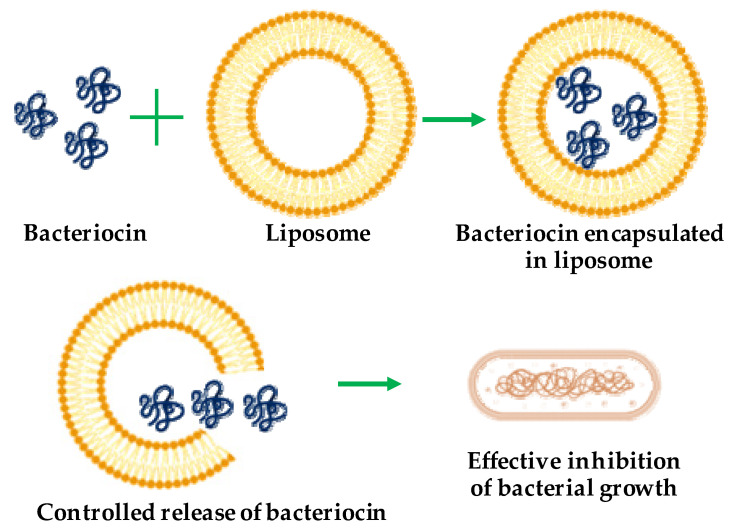
Schematic representation of bacteriocin encapsulation with liposome for antibacterial activity.

Liposomes are spherical vesicles comprising single or multiple phospholipid bilayer membranes [43]. They are non-toxic and biodegradable agents as well as suitable encapsulating materials for both hydrophilic and hydrophobic substances. The size scale of liposomes varies from micrometers to nanometers, generated via sonication or functionalization [44]. Nano-sized liposomes—called nano-liposomes—are promising vehicles for the encapsulation and delivery of different bioactive compounds such as enzymes, vitamins, and food additives, as well as the delivery of therapeutic bacteriocins to target cells [16]. Liposome encapsulation protects bacteriocins (Figure 2) from degradation caused by physicochemical environment or protease action [45], susceptibility to which is a major limitation of bacteriocins. Various studies addressing the protection of bacteriocins from protease degradation have been reported [46,47]. Liposome encapsulation also confers other advantages, such as improved stability, reduced doses in therapeutic applications, and enhanced antibacterial spectrum [10,16]. Taylor et al. [48] showed that liposomes consisting of distearoylphosphatidylcholine (PC) and distearoylphosphatidylglycerol (PG) with trapped nisin can retain approximately 70–90% of the incorporated nisin with high stability under alkaline pH and elevated temperatures (25–75 °C). Similarly, Pinilla et al. [49] showed that nanoliposomes co-encapsulated with nisin and garlic extracts became broad-spectrum antimicrobial agents against *L. monocytogenes*, *Salmonella enteritidis*, *E. coli*, and *S. aureus*. This expanded antibacterial spectrum has shown that bacteriocin could be modulated to be active on both gram-positive and -negative bacteria. The successful application of liposome-encapsulated bacteriocins depends on appropriate phospholipid bacteriocin combinations, the avoidance of adverse liposome–bacteriocin interactions, and high purity of starting materials. Such liposome-encapsulated bacteriocins have been mainly used as antibacterial substances [49] and have food-related [50] applications.

### 5.2. Chitosan

**Figure 3:**
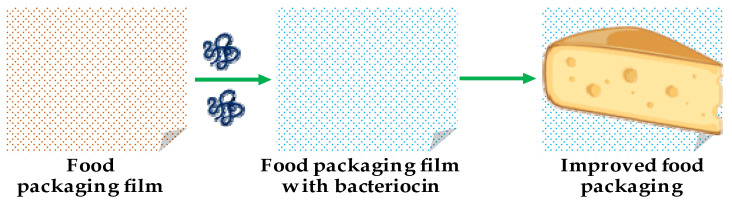
Schematic representation of improved food packaging with bacteriocin.

Chitosan is another type of nanoparticle, which is widely used with bacteriocins in various biomedical applications. Chitosan is an ideal candidate for these applications due to its non-toxic, biocompatible, and biodegradable nature [51]. Moreover, its antibacterial and anticancer activity coupled with its ability to deliver drugs to their targets is well researched [51,52]. Many researchers have used chitosan with bacteriocins to obtain a material showing synergistic antibacterial activity. For instance, Namasivayam et al. [53] reported synergistic antibacterial activity of chitosan–nanoconjugates loaded with bacteriocins against *Listeria monocytogenes*; the activity levels of these nanoconjugates were higher than those of free bacteriocins. In another study, Alireza Alishahi [54] demonstrated excellent antibacterial activity by chitosan nanoparticles loaded with nisin against *E. coli* and *S. aureus*. Moreover, nanocomposite comprised of bacteriocin and chitosan have also been used in food packaging (Figure 3). For example, Divsalar et al. [55] formed a composite film containing chitosan, cellulose, and nisin for use in packaging of ultra-filtered cheese. The composite film showed better food packaging properties than chitosan and cellulose film alone. Similar studies have been conducted using bacteriocin–chitosan nanocomposites for drug delivery [56].

### 5.3. Metallic Nanoparticles

Currently, metallic nanoparticles such as zinc, copper, silver, and gold are being studied not only for their antibacterial activity, but also for their different potential biomedical applications [12,13,57,58,59,60]. The extensive use of these nanoparticles can be attributed to their large surface area along with their positive charge, which can interact with negatively charged bacterial cell surfaces [11]. Among bacteriocin–metallic nanocomposites, Ag and Au nanoparticles are the most studied materials, showing synergistic effects in biomedical applications [10,16]. The antibacterial activity of Ag and Au nanoparticles is well reported [12]. Therefore, it is easy to understand the rationale behind the combination of bacteriocins with Ag/Au nanoparticles. This combination will not only enlarge the antibacterial spectrum, but also reduce the toxicity of nanoparticles. In this respect, Sharma et al. [32] reported enterocin-coated silver nanoparticles, which not only showed broad-spectrum inhibition against various food-borne pathogenic bacteria but also admirable non-toxicity to red blood cells, emphasizing its biocompatible nature. Pandit et al. [61] also reported the antibacterial activity of Ag–nisin nanoconjugates against *Listeria monocytogenes*, *S. aureus*, *Pseudomonas fluorescens*, *Aspergillus niger,* and *Fusarium moniliforme* associated with food spoilage. These results reveal the potential uses of bacteriocin–metal nanoconjugates in food packaging. Preet et al. [62] also reported the use of Au nanoparticles in other biomedical applications such as the co-delivery of nisin and doxorubicin to treat murine skin cancer.

### 5.4. Nanofibers

Nanofiber technology is known for its application in wound care formulations, wherein nanofibers are loaded with antimicrobials and hemostatic agents for wound healing. Bacteriocins can also be loaded with nanofibers for the same purpose [10]. The large surface area, high physical stability, and excellent encapsulation ability along with small pore size have made nanofibers the perfect nanocarriers for target-specific drug delivery [63]. Nanofiber-based bacteriocin–nanoconjugates are used as antibacterial and antiviral substances. In a recent study, Torres et al. [64] explored the efficacy of a subtilosin-loaded poly(vinyl alcohol) (PVOH) nanofiber as an antiviral agent against Herpes simplex virus type 1 (HSV-1). Subtilosin with 200 µg mL^-1^ showed a remarkable virucidal and antiviral activity against HSV-1, although the mechanistic action has not been elucidated. The PVOH-based subtilosin nanofibers with a width of 278 nm has retained subtilosin’s antibacterial efficacy, increased the loading potential of subtilosin (2.4 mg subtilosin/g of fiber) with loading efficiency of 31.6%, and demonstrated non-toxicity to human epidermal tissues. Electrospinning-based bacteriocin-nanofibers have also been studied as a drug delivery system. For instance, Ahire et al. [65] determined the activity of nisin incorporated into nanofibers prepared from poly(D,L-lactide) (PDLLA) and poly (ethylene oxide) (PEO). This combination has shown enhanced antibiofilm activity against MRSA than that by nisin alone.

### 5.5. Other Nanoplatforms

Researchers are attempting to use nanoplatforms other than liposomes, chitosan, metallic nanoparticles, or nanofibers to encapsulate or conjugate bacteriocins with nanomaterials. Niaz et al. [66] used bacteriocin-loaded nanovesicles due to their excellent antimicrobial and antibiofilm activities against food-borne pathogens. Similarly, Breukink et al. [67] evaluated the binding of nisin Z to bilayer lipid vesicles. Yadav et al. [68] assessed the interaction between nisin and vesicles synthesized using different phospholipids. Another example of nanoplatforms is solid–lipid nanoparticles (SLN), which have been used to encapsulate bacteriocins for various biomedical applications [10]. Bacteriocin-loaded SLN not only protect bacteriocins from degradation but also extend their antibacterial activity for a long period of time [69]. Phytoglycogen nanoparticles are another type of nanomaterial used to carry bacteriocins to the target site [70].

## 6. Applications

Owing to the high potential of the combined use of bacteriocins and nanomaterials, researchers have now been screening for such combinations demonstrating antibacterial and anticancer activity, with potential applications in the food and biomedical industries. In this section, the applications of the bacteriocin–nanomaterial combination will be discussed.

### 6.1. Food Applications

Over the last decade, bacteriocins have received considerable attention from the scientific community owing to their applications in the food industry as bio-preservatives either alone or in combination with other methods [4,16]. Bacteriocins are used for chemical-free preservation, extension of shelf-life, and inhibition of food-borne pathogens during farming and food-processing stages [4]. Despite these advantages, the number of bacteriocins approved by the FDA as food preservatives and antispoilage agents is limited to three (nisin, pediocin, and Micocin^®^) [11]. The limited use of bacteriocins in the food industry can be attributed to its easy degradation, electrostatic repulsion, and uncontrolled interactions with various food components. To overcome these limitations, researchers have been exploring certain synergistic approaches to increase its applications. In this regard, nanomaterials have emerged as potential partners for bacteriocins, acting in combination with bacteriocins for excellent food-related applications (Table 1).

Nanoliposomes are well-known encapsulation systems for bacteriocins because they are biocompatible, possessing both hydrophilic and hydrophobic characteristics [4,16]. Encapsulation by nanoliposomes inhibits bacteriocins from undesirable interactions with food components, controls their release, and improves their antimicrobial activity. Recently, Niaz et al. [71] demonstrated the enhanced antimicrobial activity of nisin Z against MDR food-borne pathogens after its encapsulation within a nanoliposome. In a more practical approach, Pinilla et al. [50] successfully encapsulated nisin and garlic extract into phosphatidylcholine nanoliposomes and used the system to inhibit the microbial growth of *L. monocytogenes*, *S. aureus*, *E. coli*, and *S. enteritidis* in milk. Similarly, Zou et al. [72] reported that nisin-loaded liposomal carriers inhibit two main food-borne pathogens, *L. monocytogenes* and *S. aureus*. Liposomal encapsulation can prevent the degradation of bacteriocin by the proteases in food. Recently, researchers have also attempted to use this combination in food packaging after its success as a bio-preservative. The main challenges, such as sensitivity to environmental stimuli and uncontrolled release of the antimicrobial materials in food packaging, can be overcome or minimized by using nanocarrier-mediated bacteriocin in food packaging. Boelter et al. [73] and Imran et al. [74] encapsulated nisin into nanoliposomes to produce bio-nanocomposite films for use in active food packaging. Both reports showed effective antimicrobial activity against *L. monocytogenes*. However, further studies are required to investigate the more potent food-related applications of nanoliposomes.

Apart from liposomes, chitosan is another material used in many food-related applications in combination with bacteriocins. It is used for its properties such as biodegradability, biocompatibility, and antibacterial activity [51,52]. For example, Khan et al. [75] evaluated nisin-loaded chitosan-monomethyl fumaric acid nanoparticles as a food additive. The nanocomposite significantly reduced bacterial growth in orange juice compared to the other tested samples after 48 h of incubation. Similarly, Hui et al. [76] reported better preservation of large yellow croaker with combined use of chitosan and nisin than with chitosan alone. Moreover, synergistic antimicrobial activity can be seen for nisin, lysozyme, EDTA nanoparticles, and/or ZnO nanoparticles against food-borne pathogens in minced beef [77]. In another study, Chopra et al. [83] used chitosan/carrageenan nanocapsules with nisin for enhanced antimicrobial activity. These nisin-loaded nanocarriers demonstrated excellent controlled release and synergistic antimicrobial activity in tomato juice.

Metallic nanoparticles have been used with bacteriocins in food packaging owing to their broad antibacterial spectrum and non-toxic nature at low concentration. In this regard, Song et al. [78] evaluated iron oxide nanoparticles functionalized with nisin for antimicrobial activity of *Alicyclobacillus* spp. The nanocomposite demonstrated excellent antimicrobial activity against the experimented food-spoiling microbes. Similarly, Thirumurugan et al. [79] reported that the combination of bacteriocins and gold nanoparticles displayed antimicrobial activity against food-spoiling microorganisms. In this case, antibacterial activity of the bacteriocin in combination with Au nanoparticles was better than that of the bacteriocin alone.

Nanofibers are additional nanomaterial used to encapsulate bacteriocin in food [10]. High stability and large surface area with excellent encapsulation capacity have made nanofibers good candidates for encapsulating bacteriocins. Saini et al. [80] evaluated nisin-anchored cellulose nanofibers for food packaging applications. Similarly, Soto et al. [81] encapsulated nisin into amaranth-protein-isolate:pullulan (API:PUL) nanofibers for sustained release and enhanced antimicrobial activity against *S. Typhimurium*, *L. monocytogenes*, and *L. mesenteroides* in fresh cheese and apple juice. Cui et al. [82] effectively incorporated nisin into nanofibers to prevent the growth of *L. monocytogenes* in packaged cheese.

### 6.2. Antibacterial Activity

As discussed in previous sections, the main drawbacks in using bacteriocins for antibacterial activity are their limited antibacterial spectrum and the bacterial resistance against them [10,16]. However, these problems could be resolved with the help of nanomaterials. Nanomaterials have a broad antibacterial spectrum, and the chance of bacteria gaining resistance against them is low. Therefore, bacteriocin–nanomaterial combination can be the solution to the long-standing issues with bacteriocins. Liposomes, chitosan, nanofibers, and metallic nanomaterials are mainly used in conjugation with bacteriocins for different biomedical applications (Table 2), including antibacterial activity.

In this respect, Gruskiene et al. [92] displayed antibacterial activity of iron oxide magnetic nanoparticles functionalized by nisin against gram-positive bacteria. In a more recent study, Wang et al. [84] demonstrated antibacterial activity and cytotoxicity of nisin@PEGylated MoS_2_. The nanocomposite showed antibacterial activity against both gram-positive and -negative bacteria with the mechanism of ROS production and membrane disruption strategy. The toxicity of the nanocomposite was very low, which emphasize its biomedical potential. Many researchers have encapsulated bacteriocins with liposomes to protect bacteriocins from proteases. Malheiros et al. [85] showed excellent stability and antibacterial activity of liposome-encapsulated bacteriocins against the growth of *L. monocytogenes*. Prevention of dental caries is also possible using liposome-encapsulated nisin [45]. Similarly, García-Toledo et al. [86] investigated the anti-listerial activity of pediocin encapsulated with liposome with excellent synergistic activity.

Another widely used nanomaterial that shows synergistic activities with bacteriocins is chitosan nanoparticles. Zohri et al. [87] successfully investigated the synergistic antibacterial activity of chitosan nanoparticles loaded with nisin against *S. aureus*; they exhibited two-fold higher antimicrobial activity than nisin alone. In a similar study, Namasivayam et al. [53] showed synergistic antibacterial activity of chitosan–nanoconjugates loaded with bacteriocins against *L. monocytogenes*, where a larger zone of inhibition was observed for nanoconjugates than for free bacteriocins. Hu et al. [93] demonstrated the synergistic effect of chitosan and bacteriocins against *E. coli* AW1.7 and *S. Typhimurium* in lean beef.

Metallic nanoparticles are also used with bacteriocins against bacterial infections owing to their excellent antibacterial activity [10,16]. They are commonly used because of their large surface area and positive charge, which is already discussed in previous section. Ag nanoparticles are an excellent antibacterial agent with broad antibacterial spectrum. Therefore, the combination of Ag nanoparticles and bacteriocin will not only increase antibacterial spectrum of bacteriocin but also enhance the antibacterial activity of nisin. Several researchers have followed this principle for bacteriocin–Ag nanoparticle combination toward antibacterial activity [32,61]. Au nanoparticles also showed synergistic antibacterial activity with nisin [62]. Thirumurugan et al. [79] showed the combined effect of bacteriocins and Au nanoparticles against food-spoiling microbes where the combination worked better against the microorganisms than bacteriocin alone.

In addition to their ability to inhibit bacterial growth, nanomaterials have also been used as a vehicle for targeted drug delivery. Moreover, sustained antimicrobial activity can also be achieved through this process where the effect would be highly beneficial for one week or more in many applications, such as protective textiles, medicinal products, and food packaging. Encapsulation of bacteriocin in nanofiber may give sustained delivery of bacteriocin over an extended period of time. In this regard, nanofibers are often used for targeted delivery of bacteriocin in biomedical applications. Heunis et al. [39] effectively electrospun plantaricin 423 and bacteriocin ST4SA into nanofibers, which were made from various combinations of PDLLA and PEO. Such a combination retained 88% of its original antimicrobial activity at 37 °C. The efficient capacity of the carrier matrix for bacteriocins has demonstrated by these nanofibers and therefore, the carrier maxtrix can be further used for controlled antimicrobial delivery systems. Recently, Han et al. [88] showed that the nisin released from electrospun triaxial fiber membranes showed an antimicrobial activity against *S. aureus* for up to seven days. Furthermore, the triaxial fiber membranes provided comparatively robust and more sustained antimicrobial activity than that of other forms of electrospun membranes.

### 6.3. Anticancer Activity

Beyond their typical antibacterial activities, bacteriocins show anticancer activity in combination with nanomaterials [89]. Studies have shown that bacteriocins can impact tumor growth and exhibit selective cytotoxicity toward cancer cells [94]. Nisin is a normally used bacteriocin for its anticancer activity; however, its degradation when in contact with proteases hinders its application as an *in vivo* anticancer agent [10]. Its safety profile is another issue to be resolved. Along with these drawbacks, the limited knowledge available on bacteriocin pharmacokinetics and pharmacodynamics, which are the major requirements of any anticancer drug delivery system, is an obstacle to their use as anticancer agents. In recent times, nanotechnology can be used to overcome these drawbacks. Goudarzi et al. [89] showed the cytotoxic effects of nisin-loaded PLA-PEG-PLA nanoparticles on different cancer cell lines. The results revealed that, compared to free nisin, nisin-loaded nanoparticles had synergistic cytotoxic effects on all the cancer cell lines tested. The loading efficiency of the nanoparticles was calculated as 85–90%. In another study, Au nanoparticles were used for the co-delivery of nisin and doxorubicin for their anticancer activity against murine skin cancer [62]. After evaluating the *in vivo* therapeutic effects of nanoconjugates in DMBA (7,12-Dimethylbenz(a)anthracene) induced skin carcinogenesis, mean tumor volume(s) (26.3–52.6%) reduction can be seen at the end of therapy period (~7 weeks). Moreover, all tested cytokines (TNF-α {17.39–47.89%}; TNF-β {24.52–69.88%}; NF-κβ {19.74–27.33%}; {IL-1 25.37–52.59%} and IL-10 {31.91–62.76%}) seemed to be in decline in serum levels, whereas significant increase in tissue reactive oxygen species (ROS), lipid peroxides, and superoxide dismutase activities could be seen in various treatment groups by comparing with an untreated tumor group. Along with its biocompatible nature coupled with low doses, nisin could emerge as new ray of hope as an anticancer material.

### 6.4. Other Biomedical Applications

Similar to the aforementioned applications, bacteriocins have the potential for other biomedical applications. For example, a nisin-eluting nanofiber scaffold was effectively used to treat *S. aureus*-induced skin infections in mice with no adverse effects [90]. Similarly, Au nanoparticles formulated with bacteriocins exhibited potent activity against intestinal microsporidiosis in immunocompromised mice [91]. Further studies are required to realize the full potential of nano-combinations with bacteriocins for other biomedical applications.

## 7. Conclusions and Future Perspectives

The early results of experiments examining the interface between and combination of nanomaterials and bacteriocins seem promising. The various studies discussed here have emphasized the potential biomedical applications of this platform. The problems faced with the use of bacteriocins such as protease sensitivity, limited antibacterial spectrum, high dose, and toxicity issues have been thoroughly researched, and positive results have been obtained with the nanomaterial–bacteriocin combination. However, at present, the increased use of bacteriocins in the healthcare and pharmaceutical industries is not encouraging owing to the unavailability of proper *in vivo* experimental information or clinical study data. Therefore, more *in vivo* studies are required to ensure the practicality of the nanomaterial–bacteriocin combination. Further studies are also required on the combination of other antibacterial nanomaterials such as ZnO and CuO and typical Au and Ag nanoparticles along with some polymers with bacteriocins for biomedical applications. Another area that needs more attention is the toxic nature of the combinations. Hence, researchers should focus on strategies to eradicate these limitations so that the true potential of nanomaterial–bacteriocin combinations is realized. Therefore, this nanomaterial–bacteriocin platform is in its nascent stage, but it holds promise for biomedical applications.

## Figures and Tables

**Table 1:** Various food related applications of nano-combination with bacteriocin.

Nano-Combination with Bacteriocin	Effect	Reference
Nisin Z with nanoliposome	Synergistically inhibited the growth of food-borne pathogens	[71]
Nisin into phosphatidylcholine nanoliposomes	Inhibited the microbial growth in milk	[49]
Nisin-loaded liposomal nanoparticles	Inhibition of two main food-borne pathogens, *L. monocytogenes* and *S. aureus*	[72]
Liposome-encapsulated nisin	Active food packaging	[73]
Liposome-encapsulated nisin	Synergistically act as excellent bio-preservative	[74]
Nisin-loaded chitosan-monomethyl fumaric acid nanoparticles	Excellent as a nanocarrier for food-grade antimicrobial agents and potential alternative food preservative in beverages	[75]
Chitosan with nisin	Better preservation of large yellow croaker	[76]
Nisin, lysozyme, EDTA nanoparticles, and/or ZnO	Inhibited the growth of food-borne pathogens in minced beef	[77]
Iron oxide nanoparticles functionalized with nisin	Inhibited the growth of food borne pathogens	[78]
Bacteriocin with gold nanoparticles	Synergistic growth inhibition against food spoiling microorganisms	[79]
Nisin-anchored cellulose nanofibers	Active food packaging	[80]
Nisin into amaranth-protein-isolate:pullulan (API:PUL) nanofibers	Sustained release and enhanced antimicrobial activity against food-borne pathogens in fresh cheese and apple juice	[81]
Nisin into nanofibers	Prevent the growth of *L. monocytogenes* in packaged cheese	[82]

**Table 2:** Biomedical applications of nano-combination with bacteriocins.

Nano-Combination with Bacteriocin	Effect	Reference
Nisin@PEGylated MoS_2_	Antibacterial activity against both gram-positive and -negative bacteria with low toxicity	[84]
Liposome-encapsulated nisin	Excellent stability and antimicrobial activity *L. monocytogenes*	[85]
Liposome-encapsulated nisin	Prevention of dental caries	[45]
Pediocin-encapsulated liposomes	Antilisterial activity	[86]
Chitosan nanoparticles loaded with nisin	Two-fold higher antimicrobial activity than that of nisin alone	[87]
Chitosan nanoconjugates loaded with bacteriocins	Synergistic antimicrobial activity against *L. monocytogenes*	[53]
Bacteriocin-Ag nanoparticles	Synergistic antimicrobial activity	[32,61]
Bacteriocin-Au nanoparticles	Synergistic antimicrobial activity	[79]
Plantaricin 423 and bacteriocin ST4SA into nanofibers	The combination retained its 88% original antimicrobial activity at 37 °C	[60]
Nisin electrospun into triaxial fiber membranes	Sustained release of nisin for antimicrobial activity	[88]
Nisin-loaded PLA-PEG-PLA nanoparticles	Showed cytotoxic effects on different cancer cell lines	[89]
Au nanoparticles with nisin	Delivery of the drug with anticancer activity against murine skin cancer	[62]
Nisin-eluting nanofiber scaffold	Excellent wound healing activity against *S. aureus*-induced skin infections in mice	[90]
Bacteriocin with gold nanoparticles	Showed excellent antimicrosporidial activity	[91]
